# Complete Genome Sequence of *Klebsiella pneumoniae* subsp. *pneumoniae* KP617, Coproducing OXA-232 and NDM-1 Carbapenemases, Isolated in South Korea

**DOI:** 10.1128/genomeA.01550-15

**Published:** 2016-01-14

**Authors:** Taesoo Kwon, Ji Woo Yang, Sanghyun Lee, Mi-ran Yun, Won Gi Yoo, Hwa Su Kim, Jeong-Ok Cha, Dae-Won Kim

**Affiliations:** a Division of Biosafety Evaluation and Control, Korea National Institute of Health, KCDC, Osong, Heungdeok, Cheongju, Chungbuk, Republic of Korea; b Division of Antimicrobial Resistance, Center for Infectious Diseases, Korea National Institute of Health, KCDC, Osong, Heungdeok, Cheongju, Chungbuk, Republic of Korea; c Department of Environmental Medical Biology, Chung-Ang University, Seoul, Republic of Korea

## Abstract

The prevalence of *Klebsiella pneumoniae* coproducing carbapenemase metallo-β-lactamase 1 (NDM-1) and OXA-48 has been increasing globally since 2013. The complete genome of KP617 was sequenced and assembled into a circular chromosome and two plasmids. This sequence provides the genetic background for understanding the evolution of carbapenemase genes in *K. pneumoniae* KP617.

## GENOME ANNOUNCEMENT

*Klebsiella pneumoniae* (family *Enterobacteriaceae*) is a Gram-negative, nonmotile, and facultative bacterium. *K. pneumoniae* subsp. *pneumoniae* is an opportunistic pathogen in human mucosal surfaces and can cause severe diseases, including septicemia, pneumonia, and urinary tract infections, by nosocomial routes (1, 2).

Recently, New Delhi metallo-β-lactamase 1 (NDM-1) and OXA-48 group β-lactamase carbapenemases have been responsible for carbapenem resistance in *Enterobacteriaceae* worldwide (3). Since initial reports of NDM-1 in Sweden in 2009 (4) and OXA-48 in Turkey in 2004 (5), the coproduction of NDM-1 and OXA-48 was reported in a patient after 2013 (6, 7). Here, we present the complete genome sequence of KP617, consisting of a 5.4-Mb circular chromosome and two plasmids of 273 kb and 6 kb and coproducing NDM-1 and OXA-232 carbapenemases, which were isolated in South Korea.

The genomic DNA of KP617 was extracted from a specimen from a burn patient using a Wizard genomic DNA purification kit (Promega, USA), according to the manufacturer’s instructions. Genomic DNA yield, purity, and concentration were evaluated using 0.8% agarose gel electrophoresis with a λ-HindIII digestion DNA marker and measured using a NanoDrop 1000 spectrophotometer (Thermo Fisher Scientific, USA). Three libraries were constructed for three platforms (Gnc Bio; Daejeon, Republic of Korea). First, a genomic mate-pair library with 1- to 10-kb insert sizes was constructed for an Illumina HiSeq 2500 platform. Second, PacBio RS II libraries were constructed and sequenced with 8- to 20-kb insert sizes. Third, a fosmid library with a 30-kb insert size (CopyControl fosmid library production kit; Epicentre, Madison, WI) was constructed and used as a template for physical map construction. From these platforms, 316,881,346 (32,005,015,946 bp) raw reads with Illumina HiSeq 2500 and 46,134 (421,257,386 bp) raw reads with PacBio RS II were produced. A hybrid assembly was generated using the Celera assembler (version 8.2) (8) through the PBcR pipeline (9) and confirmed with a fosmid end sequence (FES) map. The sequence gaps between contigs were filled with Sanger sequencing data after PCR amplification. The final assembly was corrected with proovread (version 2.12) (10), as the number of frameshifted genes was >5% of the called genes. The genome was composed of a 5,416,282-bp circular chromosome and two plasmids (273,628 bp and 6,141 bp, respectively). Rapid Annotations using Subsystems Technology (RAST) (11) analysis identified 5,024 putative open reading frames (ORFs) and 110 RNA genes from the circular chromosome, 342 putative ORFs from plasmid 1, and 9 putative ORFs from plasmid 2. The functional comparison of genome sequences available on the RAST server revealed *Escherichia coli* 88.1467 to be the closest neighbor of the circular chromosome (score, 527). In a comparison of KP617 and *K. pneumoniae* MGH 78578 in terms of sequence similarity, most of the functional genes of KP617 were conserved in *K. pneumoniae* MGH 78578, but 578 genes were unique (identity, ≤70%). The unique genes in KP617, such as those for β-lactamase (EC 3.5.2.6), clustered regularly interspaced short palindromic repeat (CRISPR)-associated protein family, integron, mobile element protein, phage-related protein, and transposon Tn*7* transposition proteins, show evidence for horizontal gene transfer of drug resistance genes between KP617 and other species.

### Nucleotide sequence accession numbers.

This complete shotgun genome project has been deposited in DDBJ/EMBL/GenBank under the accession numbers CP012753, CP012754, and CP012755.
